# Sociality of future outcomes moderates the effects of warmth and competence on social optimism bias

**DOI:** 10.1038/s41598-022-12816-y

**Published:** 2022-05-31

**Authors:** Mihai Dricu, Sina Ladina Jossen, Tatjana Aue

**Affiliations:** Institute of Psychology, University of Bern, Fabrikstrasse 8, 3012 Bern, Switzerland

**Keywords:** Risk factors, Human behaviour

## Abstract

People are overoptimistic about the future of those they like or admire (social optimism bias), expecting significantly more desirable than undesirable outcomes. By contrast, they are pessimistic about those they don’t like. To operationalize the (dis)like of social targets, warmth and competence are used as two universal dimensions of social perception. In this pre-registered study, we replicate previous findings while adding two new levels of complexity. First, we make the distinction between the sociality of future outcomes: “alone” outcomes (e.g., enjoying a quiet afternoon by oneself) and “social” outcomes (e.g., enjoying a vacation with the significant other). Second, we investigate the effect of attachment styles on one’s expectations for alone and social outcomes toward the social targets. In line with our hypotheses, the sociality of outcomes moderates both the additive and the multiplicative effects of the perceived warmth and competence of social targets on social optimism bias. Diverging from our hypotheses, we find that attachment anxiety and avoidance do not influence the effects of warmth and competence on social optimism bias. However, exploratory analyses reveal that attachment dimensions buffer the magnitude of social optimism bias for highly self-relevant social targets but do not impact social pessimism bias for irrelevant targets.

## Introduction

People are overoptimistic about their personal future (personal optimism bias^1,2^;) and about the future of people that they like or admire (social optimism bias^3,4^;). Accordingly, they expect significantly more desirable outcomes than negative outcomes for themselves and liked others. Previous research assessed how optimism bias^4^ relates to two universal dimensions of social perception as specified in the Stereotype Content Model (SCM), namely warmth and competence^5^. For this earlier research, four fictional characters had been devised, each being a representative of a different quadrant of the warmth × competence two-dimensional space proposed by the SCM. The participants’ task was to estimate how likely it would be that the four characters faced various desirable and undesirable events in the future. The study revealed a firm social optimism bias in that respondents anticipated desirable outcomes to be more likely than undesirable outcomes for both the warm-competent and the warm-incompetent characters. The reverse pattern was observed for the cold-incompetent character. Notably, the study matched the desirable and undesirable situations on five key characteristics, namely event valence, event frequency, event controllability, emotional intensity of the event and personal experience with the event^4^. Because earlier findings showed that each of these characteristics can significantly affect the magnitude of unrealistic optimism^2,6^, this matching procedure ruled out the possibility that confounding effects could explain the effects observed.

Our current pre-registered study builds on this previous work^4^ by adding two important modifications. First, we make the distinction between social and alone future outcomes. To our knowledge, no other research had investigated optimism bias (either personal or social) separately for alone and social outcomes. Deliberating over social interactions is inherently more complex than deliberating about single individuals because the former takes several social actors into consideration at the same time, each with their own needs and goals. The present study pits these two types of deliberations against one another to determine whether a differential optimism bias emerges. To this end, we devised a completely new set of outcomes (alone and social) matched on the same key event characteristics as in Dricu et al.^4^. Second, for the reasons outlined next, we looked at whether attachment dimensions (i.e., anxiety and avoidance) influence the perceived likelihood corresponding to social targets that vary in warmth and competence experiencing alone and social future situations. Attachment theory posits that the habitual interactions with our caregivers during childhood continue into adulthood as cognitive and emotional schemas^7,8^. These schemas influence how adults perceive themselves and others^9,10^, including the expected quality of future social encounters and alone circumstances^11–15^. For example, habitual interactions with inconsistent or overprotective caregivers in childhood predispose individuals to an anxious attachment style, consisting of low self-esteem, avoiding being alone and perpetually seeking social interaction on the assumption that only others can validate their self-worth (e.g.^14^,). This lack of independence may predispose these individuals to expect fewer positive outcomes when alone but more positive outcomes when interacting with others. By contrast, an avoidant attachment style stemming from physically or emotionally unavailable caregivers predisposes these individuals to associate self-worth with successful self-reliance, and to avoid depending on others, emotionally or otherwise (e.g.^11^,). They derive pleasure from social interactions, but these remain skin-deep. Instead, they value exploring and finding solutions to problems on their own. As such, attachment avoidance may prompt individuals to expect more positive outcomes when alone but no clear expectation toward the valence of social outcomes. Furthermore, attachment styles influence how social targets varying in warmth (e.g., friendliness and trustworthiness^12,14,15^) and competence (e.g., autonomy and self-efficacy^11,13^) are perceived and appraised (see also^16–19^). Whereas anxiously attached individuals are preoccupied with the warmth and the responsiveness of others, individuals with an avoidant attachment are particularly sensitive to the competence of others. Therefore, it is necessary to investigate whether attachment anxiety and attachment avoidance may also modulate the effects of warmth and competence of social targets on estimating their likelihood experiencing alone and social future outcomes.

Because of a lack of direct precedent in the optimism bias literature investigating alone and social outcomes separately, we based our hypotheses on attachment theory, which posits that we all strive to achieve meaningful and mutually caring relationships with others^7,20^. Because human interaction is at the forefront of our minds (on account of an availability heuristics^21^), we expected that respondents would generally rate social situations as more frequent than alone situations (Table 1, H1: main effect of sociality on likelihood estimates). Given the innate drive to seek out positive relationships and avoid negative ones^20,22^, we also hypothesized a desirability bias (i.e., optimism bias; expecting more positive than negative outcomes; Table 1, H2: main effect of valence) that would be significantly higher for social than for alone situations (Table 1, H3: interaction effect between the sociality and valence of scenarios). However, the different SCM stereotypes (factors warmth and competence) and different attachment styles (dimensions of attachment anxiety and attachment avoidance) would moderate these effects, as detailed below.

**Table 1:** List of hypotheses for social optimism.

ID	Effect	Predicted direction
H1	Sociality^a^	Social scenarios > alone scenarios
H2	Valence^a^	Desirable scenarios > undesirable scenarios
H3	Sociality × valence^a^	(Desirable > undesirable)_social_ > (Desirable > undesirable)_alone_
H4	Sociality × valence × warmth^b^	(a) Alone: (desirable > undesirable)_warm_ > (desirable = undesirable)_cold_
		(b) Social: (desirable > undesirable)_warm_ > > (desirable = undesirable)_cold_
H5	Sociality × valence × competence^b^	(a) Alone: (desirable > undesirable)_competent_ > (desirable > undesirable) _incompetent_
		(b) Social: (desirable > undesirable)_competent_ = (desirable > undesirable)_incompetent_
H6	Sociality × valence × warmth × competence^b^	(a) Warm-competent: (desirable > undesirable) _social_ > (desirable > undesirable)_alone_
		(b) Warm-incompetent: (desirable > undesirable)_social_ > (desirable > undesirable)_alone_
		(c) Cold-competent: (desirable > undesirable)_alone_ > (desirable > undesirable)_social_
		(d) Cold-incompetent: (desirable < undesirable)_social_ > (desirable < undesirable)_alone_
H7	Sociality × avoidance^a^	Highly avoidants: alone > social
H8	Sociality × avoidance × valence^a^	Highly avoidants: (desirable > undesirable)_alone_ > (desirable > undesirable)_social_
H9	Sociality × avoidance × valence × competence^a^	(a) Highly avoidants alone: (desirable > undesirable)_competent_ > > (desirable > undesirable)_incompetent_
		(b) Highly avoidants social: (desirable > undesirable)_competent_ > (desirable > undesirable)_incompetent_
H10	Sociality × anxiety^a^	Highly anxious: social > alone
H11	Sociality × anxiety × valence^a^	Highly anxious: (desirable > undesirable)_social_ > (desirable > undesirable)_alone_
H12	Sociality × anxiety × valence × warmth^a^	(a) Highly anxious social: (desirable > undesirable)_warm_ > (desirable > undesirable)_cold_
		(b) Highly anxious alone: (desirable = undesirable)_warm_ = (desirable = undesirable)_cold_

The dependent variable is the likelihood estimate (ranging from 0 to 100%). The superscripts refer to hypotheses that were not supported (a) and supported (b) by the analysis.

Social interactions represent a meeting of two minds, and one has limited control over other people’s comprehension, opinions and reactions. Therefore, the valence of future social interactions is at least partially outside of one’s direct control. However, stereotypes of warmth dictate expectations of how a social interaction would unfold because it refers to the communal predispositions of the warm interaction partner (e.g. friendliness, trustworthiness, kindness;^23–25^) and also of those around them (i.e. active help or passive facilitation from others in times of need;^5,26^). We thus hypothesized that respondents would expect the warm characters to encounter more positive (compared to negative) social situations than alone situations (Table 1, H4: the interaction effect between the sociality and valence of situations will be pronounced for warm characters (H4a) but absent or its direction reversed for cold characters (H4b)). By contrast, stereotypes of competence only refer to the perceived agentic traits of the individual (e.g. talent, intelligence, self-efficacy, confidence and self-reliance;^23–25,27^) with no expectations of active or passive interference from others^5,26^. Therefore, we hypothesized that respondents would expect competent characters to exert more control over themselves (as opposed to their social partners) and, as such, to try and boost their positive alone outcomes (compared to negative ones). We thus further hypothesized the competence dimension to exert little to no influence on social situations (Table 1, H5: an interaction effect between competence, the sociality and valence of situations will be present for alone situations (H5a) but reduced or absent for social situations (H5b)). Based on Dricu et al.^4^, we also predicted an interaction between warmth, competence, and valence. In that study, respondents anticipated significantly more desirable (than undesirable) outcomes for the two warm characters but expected more undesirable (than desirable) outcomes for the cold-incompetent character. For the current study, we predicted that the valence × warmth × competence interaction would further depend on the sociality of the events (Table 1, H6). According to the SCM, the warmth dimension is social by nature, dictating how warm individuals will engage and behave in social interactions and how those around them will respond to these attempts^23,26,28,29^. Specifically, we predicted that respondents would equally display a higher desirability bias during social situations (compared to alone) toward the warm-competent character (H6a) and the warm-incompetent character (H6b). However, the respondents would manifest a higher desirability bias during alone situations (compared to social) toward the cold-competent character (H6c), on account of their low warmth but high capacity for maximizing individual positive outcomes and minimizing individual negative outcomes. Similarly, we expected that respondents would predict significantly more undesirable than desirable events toward the cold-incompetent character, and that this would be particularly pronounced for social situations (H6d)).

Furthermore, individuals high on attachment avoidance are more sensitive to traits of competence, such as autonomy and self-efficacy (e.g.^11,13^), which are deemed more desirable than communal traits^12,15^. We thus hypothesized that respondents high on attachment avoidance would generally rate alone outcomes as likelier than social outcomes (Table 1, H7: interaction between attachment avoidance and sociality on likelihood estimates). We further predicted that respondents high on avoidance would also exhibit a higher desirability bias for alone outcomes than for social outcomes (Table 1, H8: interaction effect between sociality, valence and attachment avoidance). Lastly, we expected attachment avoidance to exacerbate the effect of perceived competence on the social optimism bias (i.e., H5). Specifically, respondents high on attachment avoidance would display a stronger bias in alone (compared to social) situations for competent characters than incompetent characters (Table 1, H9: an interaction effect between attachment avoidance, competence and the valence of situations will be present for alone situations (H9a) but reduced or absent for social situations (H9b)).

Attachment anxiety, on the other hand, makes individuals more sensitive to traits of warmth such as friendliness and trustworthiness (e.g.^13^), which are weighed more than competence traits^12,14,15^. Correspondingly, we predicted that respondents high on attachment anxiety would rate social outcomes as generally likelier than alone outcomes (Table 1, H10: interaction effect between attachment anxiety and sociality) and that they would exhibit a higher desirability bias for social situations than for alone situations (Table 1, H11: interaction effect between attachment anxiety, sociality and valence). Lastly, we expected attachment anxiety to exacerbate the effect of perceived warmth on the desirability bias. Specifically, respondents high on attachment anxiety would display a stronger desirability bias for warm (compared to cold) characters in social situations than alone situations (Table 1, H12: an interaction effect between attachment anxiety and the valence of situations will be present for social situations (H12a) but reduced or absent for alone situations (H12b)).

## Methodology

### Experimental design

The design is a 2 × 2 × 2 × 2 within-subjects design. There were two factors pertaining to the scenarios (valence: desirable and undesirable; sociality: alone and social) and two factors pertaining to the SCM characters (warmth: warm and cold; competence: competent and incompetent). Additionally, two continuous predictors pertained to the respondents (attachment anxiety and attachment avoidance).

### Stimuli

Our stimuli consisted of 48 scenarios (12 desirable alone, 12 desirable social, 12 undesirable alone, 12 undesirable social) that were balanced on sociality, valence (i.e., deviation from neutrality), frequency, controllability, and emotional intensity. To achieve this balancing, an initial pilot study was run. In a first step, we brainstormed one hundred and seventy-seven events. We then collected data on the six perceived event characteristics among one hundred and nineteen respondents from Germany (n = 95), Austria (n = 20) and Switzerland (n = 5), who were recruited on www.prolific.co (seventy-two males; range: 18–49 years old; M = 27.6 years old; SD = 6.9 years). We then determined the average scores for each of the five event characteristics separately for each of the one hundred and seventy-seven events. We excluded fifty-seven events whose 95% CI spanned the value “50” (the lower CI value was smaller than 50% and the higher CI value was larger than 50%), ending up with 120 events. Subsequently, we agreed on a final pool of 48 scenarios by using a jackknife technique, adding events in the analysis while excluding others until the events were perfectly balanced on the five event characteristics (please see Supplementary Materials, Sect. S1).

### Participants

There is no gold standard for determining a minimum sample size when designing linear mixed models^30,31^. Based on repeated simulations, several authors recommend a minimum sample size of the highest level predictor (e.g. Level 2 in a two-level linear mixed model) that ranges from 10^32^ to 50^33^ to 70^34^ to 100^35^, depending on which elements are of interest (i.e. fixed effects and standard errors; random effects and standard errors). For our study, we chose a sample size of 200 participants (our highest level predictor), which is double than the most conservative recommendation^35^. Participants were recruited via the Department of Psychology’s SONA system of a Swiss university and were given 2 ECTS credits in exchange for their participants. Inclusion criteria were: 18–30 years old, full-time students, German as mother tongue or proficiency. Self-reported mental illness served as an exclusion criterion. Data collection was conducted exclusively online and ended after 202 respondents (M = 21.98 years old, SD = 2.30 years; 133 females).

### Experimental task

Qualtrics Software (Version February 2021, Provo, UT, United States) was used to design the online survey and collect the data. The study had been approved by the local ethics committee. In accordance with the Declaration of Helsinki, all participants signed a written informed consent. Participants were informed that we aimed at testing their ability to foresee social situations. They were asked to rate the likelihood that four fictional characters (student, elderly, businessperson, alcoholic) would face the same forty-eight events (in a fully randomized fashion). The four characters were representatives of different quadrants of the warmth × competence two-dimensional space proposed by the SCM^5^. The student character (high ascribed warmth and competence) constituted the respondents’ implicit in-group^36–39^. The three remaining characters related to different types of social out-groups: (a) an alcoholic person (low ascribed warmth and competence^5^), (b) an elderly individual (high ascribed warmth, low ascribed competence^40^) and (c) a successful businessperson (low ascribed warmth, high ascribed competence^41,42^). To measure adult attachment styles, we used the short version of the Attachment Style Questionnaire (ASQ^43^;). For detailed information on how the respondents were instructed, please refer to Supplementary Materials, Sect. S3. For detailed information on the manipulation check of the characters, please see Sect. S2. We report all measures, manipulations and exclusions.

### Ethics approval

All procedures performed in studies involving human participants were approved by the ethical committee of the University of Bern, according to the standards of the Declaration of Helsinki.

### Consent to participate

All participants provided written consent.

## Analysis

### Data cleaning

Outlier identification and exclusion are identical with the procedures applied by Dricu et al.^4^ to allow for data comparison and are in line with best practices for data cleaning (^44^, see also^45,46^ for a recent implementation of the three standard deviations as data cleaning criterion). Specifically, we first determined the number of 0%, 50% and 100% likelihood estimates related to the total number of estimates at the participant level. Subsequently, we did the same at the sample level (mean across participants). Outlier participants were identified and excluded from the analysis if their answers of 0%, 50% or 100% exceeded three standard deviations of the same answers at the sample level, suggesting consistent usage of the visual analog scale in a superficial manner. In sum, we identified eight outlier participants (more details are provided in the S4 section of the Supplementary Materials).

### Design

We manipulated four within-subjects variables: two factors pertaining to the scenarios (valence: desirable and undesirable; sociality: alone and social) and two factors pertaining to the SCM characters (warmth: warm and cold; competence: competent and incompetent). Our dependent variable consisted of the participants’ likelihood ratings. We relied on linear mixed modeling (LMM) to test our hypotheses (GAMLj module in jamovi; (The jamovi project (2020). jamovi. (Version 1.6.16) [Computer Software]. Retrieved from https://www.jamovi.org.)), because it permitted the simultaneous consideration of participant- and scenario-related variance (refer to^47^ for more details on this approach, and to^48^ for its application on repeated-measures designs). Exploration and interpretation of significant interaction effects was guided by the simple effects procedure included in jamovi. The design was completely crossed, with crossed random effects for the 194 participants and the 48 situations (both Level 2 data). The Level 1 outcome was the likelihood estimate and the Level 1 predictors were the SCM dimensions warmth (warm vs. cold) and competence (competent vs. incompetent) of the character. Level 2 predictors were—as attributes of situations—the sociality of the situation (alone vs. social) and the valence of the situation (desirable vs. undesirable). Further Level 2 predictors were the participants’ (standardized) scores of attachment anxiety and attachment avoidance.

### Model selection

After a comparison of models, we selected the one that best fit our data (see Supplementary Materials, Sect. S5 for model selection^30,49^;): random intercepts for participants and scenarios; random slopes for warmth, competence and valence, with a correlated covariance structure; warmth, competence, valence, sociality and their two-way, three-way, and four-way interactions were modelled as fixed effects. Additionally, we modelled the four-way interactions between warmth, competence, valence and sociality; between warmth, valence, sociality and attachment anxiety; between competence, valence, sociality and attachment avoidance.

### Data reporting

Whenever we report interaction effects between categorical and continuous predictors, we have chosen the cut-off of +/− 1.5 SD as the midway between the population mean and the outlier cut-off. Although arbitrary, the +/− 1.5 SD cut-off has no impact on the statistical analyses, but it may provide sufficient data points to graphically display the changing patterns of behavior of respondents whose measures on the continuous predictor go from below average to average to above average.

## Results

The model comparison and selection as well as the full details of the final LMM that best fit the data can be found in the S5 section of the Supplementary Materials. This LMM did not support out hypotheses **H1**, **H2** and **H3** regarding the main effects of sociality and valence or an interaction effect between sociality and valence, respectively. Specifically, respondents rated alone and social scenarios, as well as desirable and undesirable (i.e., negative) scenarios with the same likelihood.

**Figure 1:**
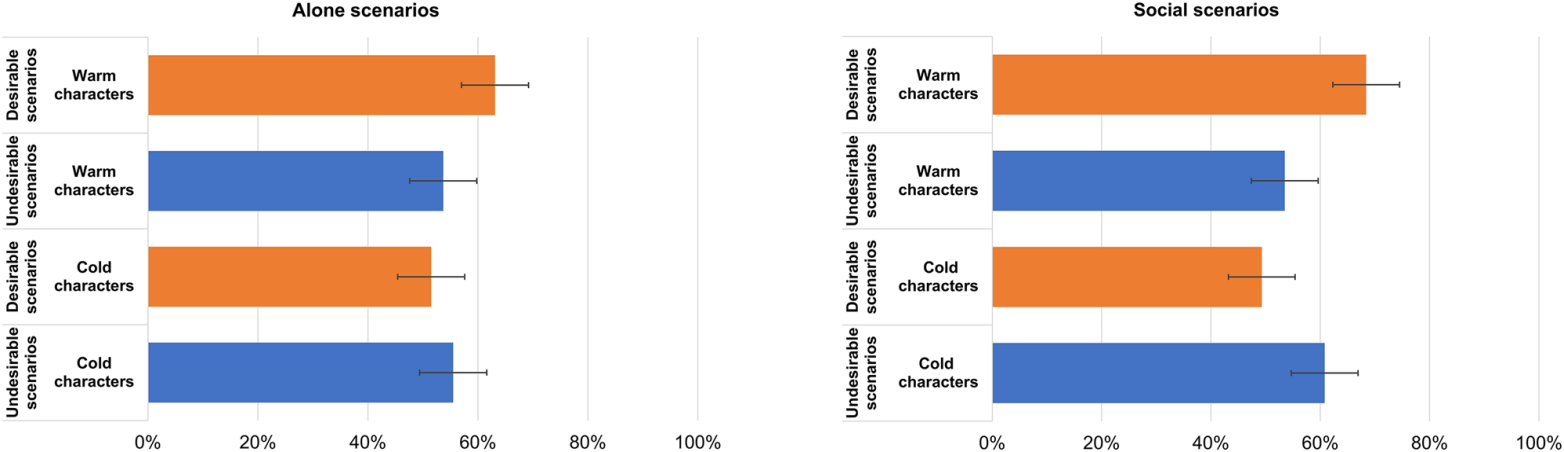
Interaction effect between warmth, valence and sociality of outcomes. The bars represent 95% confidence intervals.

However, we generally found support for our hypotheses **H4**, **H5** and **H6** concerning the moderating effect of sociality on SCM model. First, there was a three-way interaction between valence, warmth and sociality (**H4**; F (1, 36,597.0) = 183.67, *p* < 0.001). For alone situations, there was a social optimism bias for the warm characters (**H4a**; (M_diff_ = M_diff_ = 9.40%, t (44.5) = 2.15, *p* = 0.032) but no bias for the cold characters (M_diff_ = − 4.03%, t (44.5) = − 0.91, *p* = 0.361; Fig. 1). For social situations, there was a much bigger social optimism bias for the warm characters (**H4b**; (M_diff_ = M diff = 14.84%, t (44.5) = 3.37, *p* < 0.001) and a pessimism bias for the cold characters (M_diff_ = − 11.49%, t (44.5) = − 2.62, *p* = 0.009; Fig. 1).

**Figure 2:**
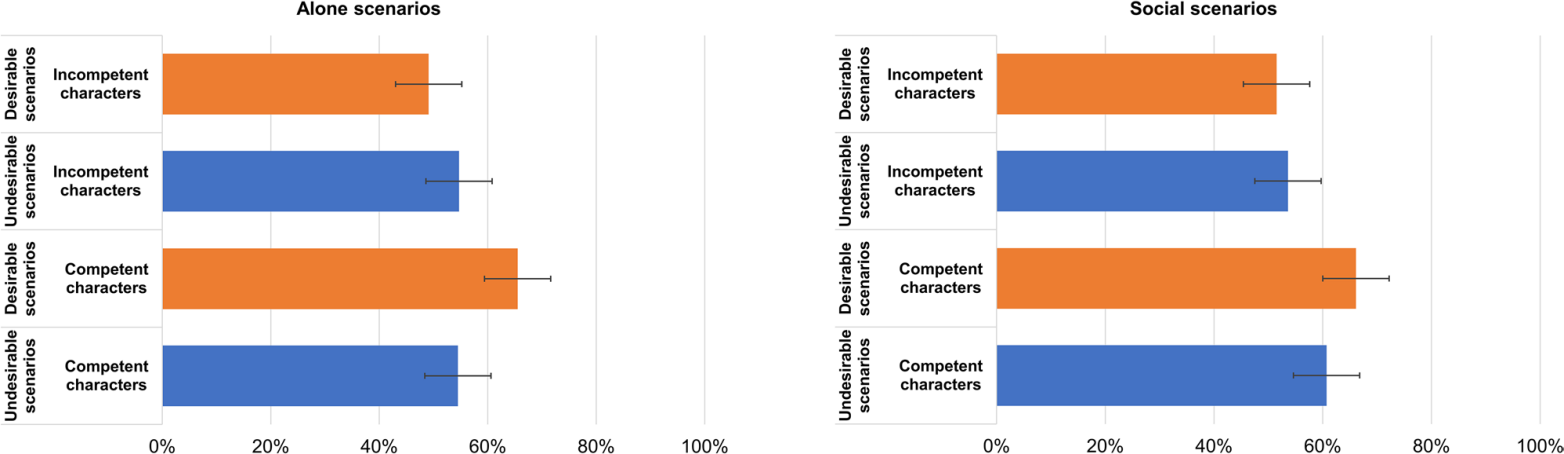
Interaction effects between competence, valence and sociality. The bars represent 95% confidence intervals.

Second, there was a three-way interaction between valence, sociality and competence (**H5**; F (1, 36,597.0) = 86.93, *p* < 0.001). For alone situations, the respondents displayed an optimism bias for competent characters (**H5a**; M_diff_ = 10.95%, t (44.5) = 2.50, *p* = 0.012) but no bias for the incompetent characters (M_diff_ = − 5.57%, t (44.5) = − 1.27, *p* = 0.204; Fig. 2). For social situations, there was no bias toward either competent characters (M_diff_ = 5.49%, t (44.5) = 1.25, *p* = 0.212) or incompetent characters (M_diff_ = − 2.15%, t (44.5) = 0.49, *p* = 0.624; Fig. 2).

**Figure 3:**
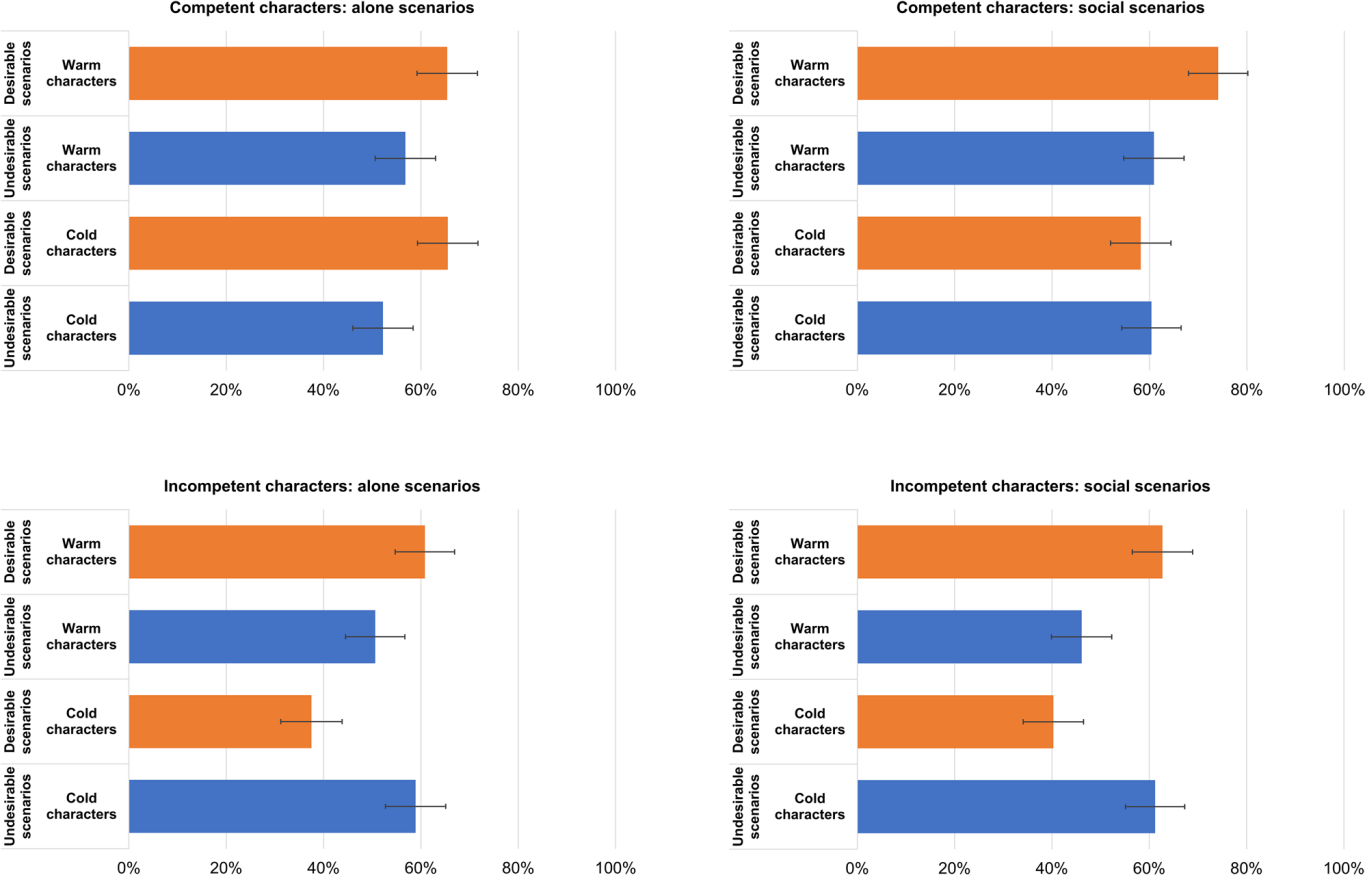
Four-way interaction between warmth, competence, valence and sociality. The bars represent 95% confidence intervals.

Finally, there was a four-way interaction between valence, sociality, warmth and competence (F (1, 36,597.0) = 54.78, *p* < 0.001) that generally supported our hypothesized directions (**H6**). For the warm-competent character (**H6a**), there was social optimism bias during social situations (M_diff_ = 13.13%, t (23.9) = 3.54, *p* = 0.002) but not alone situations (M_diff_ = 8.61%, t (22.9) = 1.71, *p* = 0.101; Fig. 3). Similarly, respondents manifested an optimism bias toward the warm-incompetent character (**H6b**) during social situations (M_diff_ = 16.55%. t (23.9) = 4.46, *p* < 0.001) but no bias during alone situations (M_diff_ = 10.20%, t (22.9) = 2.02, *p* = 0.055; Fig. 3). For the cold-competent character (**H6c**), there was an optimism bias during alone scenarios (M_diff_ = 13.29%, t (22.9) = 2.64, *p* = 0.015) but no bias during alone scenarios (M_diff_ = − 2.14%, t (23.9) = − 0.58, *p* = 0.569; Fig. 3). By contrast, respondents manifested a pessimism bias during both social (M_diff_ = − 20.85%, t (23.9) = − 5.62, *p* < 0.001) and alone scenarios (M_diff_ = − 21.34%, t (22.9) = − 4.23, *p* < 0.001; Fig. 3) toward the cold-incompetent character (**H6d**).

None of our hypotheses about attachment anxiety and attachment avoidance were supported. There was no interaction effect between attachment avoidance and sociality (**H7**; F (1,36,410.0) = 0.21, *p* = 0.649), meaning that respondents rated alone and social events irrespective of the level of attachment avoidance. Furthermore, there was no interaction between attachment avoidance, sociality and valence (**H8**; F (1,36,410.0) = 0.22, *p* = 0.635), suggesting that there was no optimistic or pessimistic bias for either alone or social events, regardless of the level of attachment avoidance. Lastly, there was no interaction between attachment avoidance, sociality, valence and competence (**H9**; F (1,34,610.0) = 0.14, *p* = 0.707). However, there was a significant three-way interaction between attachment avoidance, valence and competence that was not predicted (F 1,36,406.0) = 6.04, *p* = 0.014. Specifically, there was no bias for incompetent characters, regardless of the level of attachment avoidance (Mean − 1.5 standard deviation (SD): t (46.4) = − 1.11, *p* = 0.265, Mean: t (46.4) = − 1.23, *p* = 0.218; Mean + 1.5 SD: t (46.4) = − 1.26, *p* = 0.207). However, there was a significant optimism bias for competent characters whose magnitude decreased as attachment avoidance increased (Mean − 1.5 SD: t (46.4) = 3.14, *p* = 0.002; Mean: t (46.4) = 2.63, *p* = 0.009; Mean + 1.5 SD: t (46.4) = 1.92, *p* = 0.055). This goes against the predicted direction in **H9**. There was also no interaction effect between attachment anxiety and sociality (**H10**; F (1,34,610.0) = 0.29, *p* = 0.592), meaning that respondents rated alone and social events irrespective of the level of attachment anxiety. Furthermore, there was no interaction between attachment anxiety, sociality and valence (**H11**; F (1,34,610.0) = 0.36, *p* = 0.546), suggesting that there was no optimism or pessimism bias for either alone or social events, regardless of the reported level of attachment anxiety. Lastly, there was no interaction between attachment anxiety, sociality, valence and warmth (**H12**; F (1,34,610.0) = 0.32, *p* = 0.574).

### Exploratory analyses

**Figure 4:**
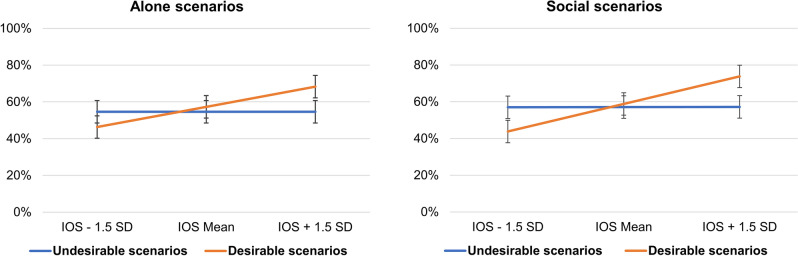
Interaction effect between scores of Inclusion of Others in Self (IOS), valence and sociality. The bars represent 95% confidence intervals.

To investigate potential reasons for not finding general support for our hypotheses regarding attachment dimensions (**H7**–**H12**), we embarked on an exploratory analysis. Specifically, we asked whether other social cognition factors than warmth and competence might modulate the effects on attachment anxiety and avoidance on optimism bias about others. Because the mental schemes about others triggered by an individual’s attachment style are confined to how responsive others would be to their own needs, social cognition in individuals with an unsecure attachment style is egocentric, revolving around the question of how relevant others are to their own needs and fears^10,13,50–52^. By contrast, stereotypical thinking along dimensions of warmth and competence are cognitions and emotions widely held by society that transcend the individual needs and fears of the members of that society^24,25,27^. A possible reason why our hypotheses about attachment avoidance and anxiety were not supported could be because dimensions of warmth and competence are not relevant enough for the respondent in order for the attachment styles to manifest. To test this possibility, we ran an exploratory analysis replacing warmth and competence with scores of the Inclusion of Others in the Self Scale (IOS Scale; see S6 section of the Supplementary Materials), which indexes the degree of relevance of others to oneself^53,54^. This analysis showed that attachment anxiety and avoidance interact with the degree of self-relevance of the social target to modulate the magnitude of the optimism bias. First, there was an interaction between valence and IOS (F (1,30,199.08) = 1062.03, *p* < 0.001) with respondents being generally pessimistic toward social targets with low self-relevance (Mean IOS − 1.5 SD: M_diff_ = − 10.70%; t (45.96) = − 3.4, *p* < 0.001) but optimistic towards social targets with high self-relevance (Mean IOS + 1.5 SD: M_diff_ = 15.20%; t (45.96) = 4.82, *p* < 0.001). However, this effect was qualified by further interactions between valence, IOS and sociality of events (F (1,36,796.7) = 28.05, *p* < 0.001; Fig. 4) as well as by three-way interactions with attachment anxiety (F (1,28,958.5) = 7.86, *p* = 0.005) and attachment avoidance (F (1,31,615.8) = 4.07, *p* = 0.044), respectively. Respondents were overall more optimistically biased toward social targets at high scores of IOS (Mean IOS + 1.5 SD) when the outcomes were social (M_diff_ = 16.60%, t (45.96) = 3.76, *p* < 0.001) than alone (M_diff_ = 13.70%, t (45.96) = 3.1, *p* = 0.002; Fig. 4).

**Figure 5:**
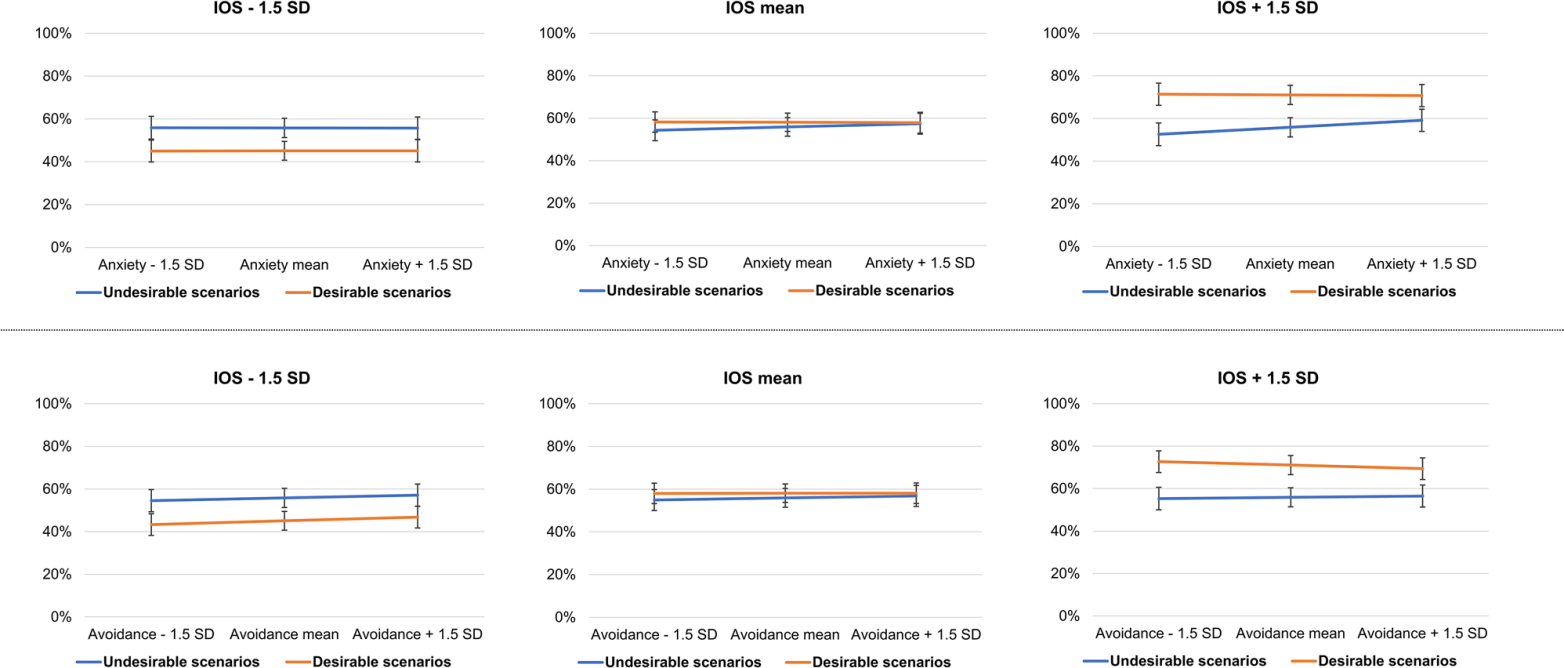
Interaction effect between IOS, valence and attachment dimensions. The bars represent 95% confidence intervals.

While the attachment dimensions did not affect the levels of pessimism at low IOS, they buffered the levels of optimism at high IOS. Specifically, respondents were progressively less optimistically biased toward highly self-relevant social targets (Mean IOS + 1.5 SD) as attachment anxiety increased from very low (Mean − 1.5 SD, t (45.96) = 5.63, *p* < 0.001) to average (t (45.96) = 4.82, *p* < 0.001) to very high (Mean + 1.5 SD, t (45.96) = 3.48, *p* < 0.001, Fig. 5) as well as when attachment avoidance increased from very low (Mean − 1.5 SD, t (45.96) = 5.22, *p* < 0.001) to average (t (45.96) = 4.82, *p* < 0.001) to very high (Mean + 1.5 SD, t (45.96) = 3.88, *p* < 0.001; Fig. 5). For attachment anxiety, the pattern was driven by an increasing slope for negative events (Mean IOS + 1.5 SD, t (190.8) = 3.59, *p* = 0.001; Fig. 5) but not for positive events (Mean IOS + 1.5 SD, t (190.8) = − 0.34 *p* = 0.734; Fig. 5). These patterns suggest that social optimism bias is maximal among the respondents with a secure attachment style (low attachment anxiety/avoidance) that identify the most with the social targets.

## Discussion

This preregistered study builds upon previous work (e.g.^3,4,55^), which showed that people are overly optimistic about the future of those that they like or admire (social optimism bias), expecting significantly more desirable outcomes than undesirable outcomes, but that they are pessimistic about the future of those they dislike (social pessimism bias). To operationalize (dis)liking, we used warmth and competence, two universal dimensions of social perception^5,25^. In the present study, we replicated previous findings with an entirely different set of stimuli while also adding two new levels of complexity. First, we made the distinction between the sociality of future outcomes: “alone” outcomes (e.g., enjoying a quiet afternoon by oneself) and “social” outcomes (e.g., meeting an old friend by accident on the street). Second, we investigated the effect of attachment styles on one’s expectations of alone and social outcomes for social targets. To our knowledge, no other study has previously explored the influence of a future outcome’s sociality and a respondent’s attachment styles on optimistic expectancies.

In line with our hypotheses, we find that the sociality of outcomes moderates both the additive and the multiplicative effects of the perceived warmth and competence of social targets on social optimism bias. Specifically, our previous finding for warm characters is driven exclusively by social outcomes and the respondents are no longer biased during alone outcomes (we note that there was a trend toward optimism bias for the warm-incompetent character). By contrast, respondents manifested an optimism bias toward the cold-competent character during alone scenarios but no bias during social situations. Finally, respondents manifested a pessimism bias toward the cold-incompetent character regardless of the sociality of the outcomes.

These findings expand on the current body of social perception and serve as further validation of the SCM model. The univalent negative stereotypes (cold and incompetent) of social targets are particularly robust in the face of everyday situations, in line with the dehumanized perception of certain groups of people such as the homeless and drug addicts (see^56^), stemming from reduced activation in the otherwise normally recruited social-cognition neural network (^57^; see also^58^). While our data seem to suggest that communal (warmth) and agentic (competence) stereotypes commonly held by a society are very robust and transcend individual attachment-based mental schemes, future research should investigate whether the attributed communal trait morality and the attributed agentic trait assertiveness (cf.^59^) are equally robust in this regard. Because we had not assessed these perceptual facets in the current study, we were not able to perform corresponding analyses on the data at hand.

That the optimism bias toward warm characters was driven exclusively by social situations and that the pessimism bias toward the cold-incompetent character was relentless are both consistent with SCM observations for peer attitudes towards these social group members. The warmth dimension of the SCM is a social dimension, indexing how friendly, trustworthy and empathetic an individual is perceived by their peers^23,26,28,29^. Because of this, SCM predicts that warm characters are either actively helped or passively supported in their daily activities by others, whereas cold characters are either actively harmed or passively neglected^5^. In other words, the SCM makes predictions about how warm and cold characters will be acknowledged and received by their peers in social interactions. By contrast, the competence dimension is an agentic dimension that is orthogonal to warmth^23,26,28,29^. While being warm is other-profitable, being competent is only self-profitable^5^. An extension of this is that competent individuals may not have a lot of influence over others (not as much as warm individuals) but they are intelligent, knowledgeable, or skillful enough to pursue and attain individual goals. Consequently, competent people are most apt to maximize positive alone outcomes and minimize negative alone outcomes. Although cold-competent characters are penalized in social situations on account of their low warmth, they may prevail in alone situations on account of their skills and competence. An open question remains as to why the warm-competent character was not also optimistically perceived in alone situations on account of the same high competence. A possible explanation may come again from the SCM, which posits that warmth and competence are not only orthogonal but may also serve as a buffer to one other^42,60,61^. On the one hand, there is a trade-off between warmth and competence: when an individual makes active attempts to increase their competence (warmth) reputation it leads inevitably to losing points for their warmth (competence) reputation^60^. On the other hand, there is a ceiling effect to how competent a warm person can be perceived by others (and how warm a competent person can appear^61^;). The warm-competent character may therefore be held back in alone situations by their high warmth levels.

Interestingly, the pessimism bias for the cold-incompetent character remained robust regardless of the sociality of situations (post-hoc tests revealed no difference in the magnitude of the pessimism bias for alone and social situations). Considering the arguments presented above, the unique combination of low warmth and low competence makes these individuals particularly vulnerable during both alone and social situations. This may be because respondents expect cold-incompetent characters to be unable to minimize negative alone outcomes through personal actions like the cold-competent character but also unable to maximize positive social outcomes through the facilitatory interventions of others like the warm-incompetent character.

We further hypothesized that attachment anxiety and attachment avoidance would moderate the effects of warmth and competence, respectively, on the direction and magnitude of social optimism bias. High attachment anxiety (avoidance) makes individuals more vigilant toward warmth (competence) traits in others^11–15^ so we expected that attachment dimensions would exacerbate the effects of warmth and competence. However, our hypotheses were not supported by the findings, suggesting that factors other than attachment styles (if any) may exacerbate the influence of warmth and competence in social perception and/or attachment styles modulate other social perception attributes than warmth and competence. Our exploratory analysis replacing warmth and competence with scores of IOS, which indexes the degree of relevance of others to oneself, seems to indicate that the dimensions of warmth and competence per se are not sufficiently relevant for the respondent in order for the attachment anxiety and avoidance to manifest. Instead, insecure attachment styles may predispose individuals towards an egocentric social cognition^10,13,50–52^ making them more sensitive toward the relevance of others to their own needs and fears. This self-relevance may transcend the cognitions and emotions widely held by the members of a society toward certain social targets, which the dimensions of warmth and competence tap into^24,25,27^. This exploratory analysis suggests that attachment anxiety and avoidance modulate the magnitude of the optimism bias displayed toward other social groups depending on the degree of their relevance to the self. On average, respondents were pessimistically biased toward social targets with low self-relevance (i.e., low scores of IOS) and optimistically biased towards social targets with high self-relevance. However, both attachment anxiety and attachment avoidance modulated this relationship: while attachment dimensions did not affect the levels of pessimism, they buffered the levels of optimism. Specifically, respondents with high and very high levels of attachment anxiety or avoidance were significantly less optimistic toward highly self-relevant social targets. One explanation for this pattern of findings is that individuals with an insecure attachment style may project their own insecurities^62–64^ onto social targets with whom they identify highly, leading respondents to manifest a significantly reduced optimism bias toward these targets. However, we encourage future studies to actively test for this possibility and the other findings from our exploratory analysis. We acknowledge that there may be a lot to uncover regarding how attachment dimensions affect social cognition, including optimism bias toward highly-relevant social targets. At the very least, the lack of support for our hypotheses regarding the modulating effect of attachment dimensions on warmth and competence strongly suggests that communal and agentic stereotypes commonly held by a society are very robust and transcend individual attachment-based mental schemes.

In conclusion, our study shows that the sociality of future outcomes is an important factor to consider when investigating (social) optimism bias, tapping into distinct dimensions of social targets, i.e., social outcomes tap into the warmth dimension and alone outcomes tap into the competence dimension. Furthermore, insecure attachment styles can also play a significant role in social optimism bias. Traditionally, social optimism bias has been investigated toward social targets with various degrees of closeness to the respondents, such as vocational similarity^65^ or close friendships^37,66–70^. Our study shows that the magnitude of social optimism toward highly self-relevant others is buffered by attachment insecurity while the pessimism bias towards irrelevant others stands unaffected. Lastly, individual lines of research have shown that both securely-attached individuals (e.g.^71–73^) and highly optimistic people (e.g.^55,74,75^) have the highest levels of subjective well-being and physical health and are best equipped to deal with adverse situations in life. Our research is the first to link these two independent lines of findings by suggesting that securely-attached individuals (i.e. low anxiety/low avoidance) and optimistically biased individuals may be part of the same population.

## Supplementary Information

Supplementary Information.

## Data Availability

The data that support the findings of this study are openly available in Open Science Framework at http://doi.org/10.17605/OSF.IO/M95PU.
