# Supplementary material for: Sociality of future outcomes moderates the effects of warmth and competence on social optimism bias
Source: Sci Rep. 2022 May 31;12:9060. doi: 10.1038/s41598-022-12816-y (PMC9156740; doi:10.1038/s41598-022-12816-y)
Supplement: Supplementary file 1 — Supplementary Information. [file 41598_2022_12816_MOESM1_ESM.docx]

**Supplementary Materials**

## S1: Manipulation check of the events

The success of our task assessing optimism bias depended on future outcomes with a balanced range of characteristics. To make sure that our stimuli only differed on the desired characteristics, while keeping the others constant, we conducted separate linear mixed models with factors **sociality** (two levels: alone and social) and **valence** (two levels: desirable and undesirable) on each of the five event characteristics: *perceived socialit*y, *valence*, *frequency*, *controllability*, and *emotional intensity*.

The linear mixed model with *perceived sociality* as the dependent variable revealed, as intended, a main effect of sociality (F (1,44) = 343.94, p < .001) but not a main effect of valence (F (1,44) = .53, p = .468) or an interaction between valence and sociality (F (1,44) = 1.66, p =.205). Specifically, alone events (M = 37.8, SE = 1.61) were rated significantly lower on sociality than social events (M = 76.1, SE = 1.61). The linear mixed model with *perceived valence* as the dependent variable yielded, as intended, a main effect of valence (F (1,44) = 302.31, p < .001) but not a main effect of sociality (F (1,44) = .13, p = .717) or an interaction between valence and sociality (F (1,44) = .08, p =.779). Specifically, desirable events (M = 74.7, SE = 2.10) were rated significantly higher on valence than undesirable events (M = 23.4, SE = 2.10). The linear mixed model with *perceived frequency* in the general population as the dependent variable revealed, as intended, no main effects of valence (F (1,44) = 1.78, p = .189) or sociality (F (1,44) = .60, p = .441) nor an interaction between valence and sociality (F (1,44) = .09, p =.769). The linear mixed model with *perceived controllability* as the dependent variable yielded, as intended, no main effects of valence (F (1,44) = 1.10, p = .300) or sociality (F (1,44) = 2.37, p = .131) nor an interaction between valence and sociality (F (1,44) = .276, p =.602). Finally, the linear mixed model with *perceived emotional intensity* as the dependent variable revealed, as intended, no main effects of valence (F (1,44) = 1.72, p = .197) or sociality (F (1,44) = 3.41, p = .071) nor an interaction between valence and sociality (F (1,44) = .150, p =.700). Furthermore, the study’s (N=202) perceived sociality (Pearson’s r = .95, p < .001), valence (Pearson’s r = .97, p < .001), frequency (Pearson’s r = .93, p < .001), controllability (Pearson’s r = .94, p < .001) and emotional intensity (Pearson’s r = .96, p < .001) for the different events correlated very strongly with the same values in the pilot (N =119), suggesting very high agreement between the two samples.

To determine whether the events were balanced on valence (deviation from neutrality, i.e., hypothetical “0” middle value on the visual analog scale), we performed the following analysis: Because the raw valence scores of undesirable events and desirable events ranged from “-50” to “0” and “0” to “50”, respectively, we gave the raw scores of the undesirable values a positive sign. Afterwards, we compared these absolute valence scores of desirable vs undesirable events by performing a linear mixed model analysis on them. The analysis revealed that the absolute values of the desirable (M = 24.7, SD = 18.5) and undesirable events (M = 26.6, SD = 18.3) did not statistically differ (F (1,44) = 0.408, p = .526), indicating that the desirable and undesirable events were perceived to be equidistant from a hypothetical “0” middle value denoting valence neutrality.

In summary, the results presented above suggest that the designated desirable and undesirable alone and social life situations were matched on the different characteristics under investigation.

| **Scenario** | **Perceived sociality** | **Perceived valence** | **Perceived frequency** | **Perceived controllability** | **Perceived intensity** |
| --- | --- | --- | --- | --- | --- |
| Paying bills. | -6 | -12 | 83 | 64 | 32 |
| Being lonely in old age. | -13 | -39 | 59 | 50 | 82 |
| Carrying the trash outside. | -10 | -1 | 80 | 79 | 13 |
| Finding rotten food in the refrigerator. | -11 | -29 | 51 | 78 | 32 |
| Not having access to a power socket when traveling. | -8 | -16 | 48 | 27 | 34 |
| Getting food poisoning. | -19 | -40 | 26 | 37 | 50 |
| Experiencing a huge financial loss. | 1 | -39 | 43 | 50 | 80 |
| Finding food past its expiration date in the cupboard. | -7 | -21 | 66 | 77 | 29 |
| Being bitten by a dog. | -2 | -39 | 23 | 30 | 68 |
| Being alone on one's birthday. | -14 | -34 | 40 | 55 | 78 |
| Getting a sunburn. | -29 | -29 | 61 | 78 | 32 |
| Getting stuck in an elevator. | -3 | -32 | 18 | 13 | 63 |
| Saying something that upsets a friend. | 30 | -27 | 56 | 66 | 69 |
| A neighbor listening to music too loudly when ones wants to sleep. | 26 | -28 | 45 | 32 | 56 |
| Being lied to by one's partner. | 25 | -40 | 60 | 28 | 86 |
| Having to work with someone one doesn't like. | 25 | -28 | 67 | 35 | 62 |
| Waiting fifteen minutes for a friend who is late. | 24 | -18 | 58 | 26 | 45 |
| The airplane one is waiting to board is ten minutes late. | 29 | -16 | 55 | 12 | 41 |
| Being misunderstood when trying to explain one's point of view. | 25 | -24 | 60 | 46 | 63 |
| Being responsible for someone's emotional suffering. | 29 | -38 | 46 | 66 | 84 |
| Being mistaken for another person by someone. | 20 | -4 | 37 | 18 | 33 |
| Arguing with a friend | 31 | -31 | 54 | 59 | 77 |
| Someone is making fun of you. | 16 | -33 | 48 | 37 | 75 |
| Accidentally pouring a drink over a friend. | 26 | -21 | 39 | 49 | 46 |
| Getting five francs interest from the bank at the end of the month. | -21 | 16 | 42 | 43 | 32 |
| Getting a haircut. | -10 | 20 | 74 | 76 | 37 |
| Being able to pay in a store with a credit card | -12 | 11 | 73 | 69 | 19 |
| Living the lifestyle one has always wanted. | -6 | 37 | 51 | 69 | 71 |
| Seeing a shooting star. | -23 | 30 | 35 | 19 | 57 |
| Learning a new language. | -22 | 29 | 54 | 83 | 39 |
| Win an all-inclusive trip for one person. | -16 | 35 | 14 | 12 | 70 |
| Make a purchase of more than 50 francs for one's own pleasure. | -22 | 18 | 64 | 80 | 45 |
| Living a healthy and active life until death. | -13 | 36 | 46 | 77 | 57 |
| Finding a free seat on the bus. | 11 | 18 | 73 | 34 | 25 |
| Seeing an interesting offer in the supermarket. | -12 | 18 | 69 | 41 | 39 |
| Enjoying a quiet afternoon alone. | -27 | 29 | 64 | 76 | 44 |
| Meeting a colleague at the cinema. | 26 | 21 | 38 | 21 | 51 |
| Being asked for directions by someone on the street. | 25 | 9 | 48 | 35 | 25 |
| Saying hello to a neighbor in the stairwell. | 30 | 22 | 71 | 68 | 36 |
| Having relatives greet you in a friendly way at a family gathering. | 30 | 32 | 77 | 53 | 64 |
| Cooking vegetarian food for friends. | 28 | 26 | 54 | 83 | 40 |
| Meeting an old friend by chance on the street. | 26 | 22 | 54 | 23 | 60 |
| A new neighbor comes over to introduce themselves. | 26 | 20 | 41 | 29 | 42 |
| Going to a party with friends and having fun. | 32 | 35 | 70 | 73 | 68 |
| Seeing two people kissing on the street. | 26 | 11 | 78 | 20 | 40 |
| Going on vacation with one's partner. | 33 | 36 | 66 | 75 | 73 |
| Donating money to a person in need. | 18 | 29 | 44 | 84 | 57 |
| Getting a birthday gift from friends. | 20 | 35 | 78 | 36 | 71 |

**Supplementary Table S1.** Average ratings for each of the five event characteristics. Perceived valence was rated on a continuous scale from -50 (very negative/undesirable to 50 (very positive/desirable). All other characteristics were rated on a continuous scale from 0 (very low) to 100 (very high).

## S2: Manipulation check of the SCM characters.

To determine whether the participants identified with the student characters as hypothesized and, thus, whether the in-group – out-group manipulation worked, we analyzed the ratings of the Inclusion of Other in the Self Scale. A linear mixed model with **respondent** as a cluster factor, and the factor **character** (four levels: alcoholic, elderly, businessperson, student) on scores of the *IOS* measure yielded significant differences in how much participants identified with each character (F (3,579) = 272, p < .001). Pairwise comparisons using the Bonferroni correction revealed that all characters were rated significantly different from each other (all *p*s < .001) with the exception of the elderly and the businessperson (p = .505): the participants identified the most with the student character (*M* = 5.41, SE = .09), followed by the elderly character (*M* = 2.99, SE = .09), the businessperson (*M* = 2.76, SE = .09) and the alcoholic character (*M* = 1.71, SE = .09).

To provide a quick post-hoc check on whether the assumptions of designated warmth and competence would hold in our sample, we analyzed the scores of perceived warmth and competence of each of the four characters. A linear mixed model with **respondent** as a cluster factor, and the factors **character** (four levels: alcoholic, elderly, businessperson, student) and **reference point** (two levels: self and society) on scores of perceived *warmth* (**Supplementary Figure S2.1**) showed main effects of character (F (3,1351) = 846.4, p < .001) and reference (F (3,1351) = 19.8, p < .001) and an interaction between character and reference (F (3,1351) = 25.1, p < .001). Pairwise comparisons using the Bonferroni correction revealed that all characters were rated significantly different from each other (all *p*s < .001): the elderly character (*M* = 5.92, SE = .06), the student *M* = 5.33, SE = .06), the businessperson *M* = 4.31, SE = .06) and the alcoholic (*M* = 3.07, SE = .06). Overall, respondents rated all characters as warmer (*M* = 4.81, SE = .04) than they assumed society at large views them (*M* = 4.61, SE = .04). This was entirely driven by the evaluation of the alcoholic character: whereas respondents evaluated the three remaining characters identically, regardless of the reference point (the elderly character (self: *M* = 5.89; others: *M* = 5.96), the student character (self: *M* = 5.50; others: *M* = 5.56) and the businessperson (self: *M* = 4.34; others: *M* = 4.27)). Respondents evaluated the alcoholic character warmer (self: *M* = 3.50) than they believed the society at large evaluated them (others: *M* = 2.65). This could be due to social desirability effects, where the alcoholic is a highly contentious social category.


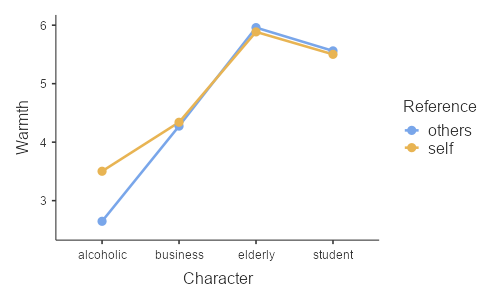


**Supplementary Figure S2.1.** Manipulation check of the SCM characters; warmth rating**.**

A linear mixed model with **respondent** as a cluster factor, and the factors **character** (four levels: alcoholic, elderly, businessperson, student) and **reference point** (two levels: self and society) on scores of perceived *competence* (**Supplementary Figure S2.2)** showed main effects of character (F (3,1351) = 1628.4, p < .001) and reference (F (3,1351) = 12.4, p < .001) and an interaction between character and reference (F (3,1351) = 29.8, p < .001).

Pairwise comparisons using the Bonferroni correction revealed that all characters were rated significantly different from each other (all *p*s < .001): the businessperson character (*M* = 6.19, SE = .04), the student (*M* = 5.60, SE = .04), the elderly (*M* = 4.75, SE = .04) and the alcoholic (*M* = 2.77, SE = .04). Overall, respondents rated all characters as more competent (*M* = 4.89, SE = .03) than they assumed society at large views them (*M* = 4.76, SE = .03). As with warmth ratings, this was entirely driven by the evaluation of the alcoholic character: whereas respondents evaluated three characters identically, regardless of the reference point (the businessperson (self: *M* = 6.12; others: *M* = 6.26), the student character (self: *M* = 5.52; others: *M* = 5.68) and the elderly character (self: *M* = 4.81; others: *M* = 4.69), respondents evaluated the alcoholic character as more competent (self: *M* = 3.12) than they believed the society at large evaluated them (others: *M* = 2.41). This could be also due to social desirability effects, where the alcoholic is a highly contentious social category.


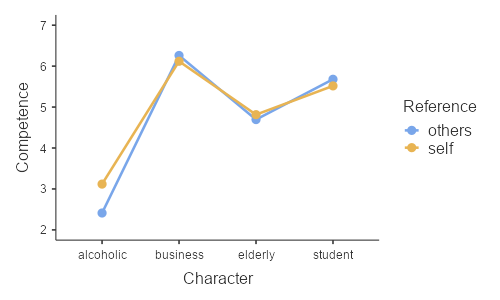


**Supplementary Figure S2.2.** Manipulation check of the SCM characters; competence rating**.**

# S3. Experimental task

The experimental task required participants to give likelihood estimates for four fictional characters experiencing the same forty-eight events (in a fully randomized fashion). These four characters were chosen to reflect each of the quadrants of the two-dimensional space of warmth and competence of the SCM model ^1^. A student character served as the implicit in-group for our participants ^2-5^, high on both warmth and competence. Three other characters were created to serve as out-groups: a) an alcoholic person (low on both warmth and competence ^1^), b) an elderly person (high on warmth but low on competence ^6^) and c) a successful businessperson (high on competence but low on warmth ^7,8^).

Participants were told to think of each character as a representative member of their social group (i.e. “This is a typical [student / elderly person / businessperson / alcoholic person]”). To ease the participants’ task, we created still animations to illustrate each character in each scenario. Four standalone animated characters reflecting the SCM stereotypes were first designed in line with ^9^. Additionally, one hundred and ninety-two situations were created to reflect each character in each of the forty-eight events discussed above, with one set of events per character. All characters, backgrounds and scenarios were designed using the game The Sims 4 (Electronic Arts, California, USA). To avoid possible gender influences, we created a male and a female version for every stereotype × scenario combination. Female participants saw female animated characters and male participants viewed male animated characters.

The experiment consisted of four blocks. The first block started by asking participants to familiarize themselves with the four characters and informed them of the specific task, i.e. providing likelihood estimates for each of the four SCM characters experiencing each of the forty-eight scenarios. This familiarization period included three examples, after which the real task began: providing likelihood estimates for a total of one hundred and ninety-two scenarios, which were displayed in a randomized order and without interruptions. Each trial/scenario was displayed for 10s and showed a single still animation of the target character in the upper part of the screen, above a one-sentence description of the target situation. At the bottom of the screen, there was a visual analogue scale (VAS) with which the participants selected a percentage from 0% (left side) to 100% (right side). The VAS always started with the slider on the middle position (50%). Participants had 10s to decide: if the time window passed and the slider of the VAS had not been moved, a reminder was shown on the top of the screen that one cannot proceed to the next trial until a decision is made.

The second block of the experiment included two sets of evaluations that were randomly presented. The first set prompted participants to rate the four SCM characters on the Inclusion of Other in the Self (IOS) scale ^10,11^. The IOS scale consists of seven pairs of diagram-like overlapping circles on a continuum from a lesser to a greater overlap. The degree of overlap depicted by each of the individual pairs represents a degree of interconnectedness (on a scale from 1 (“not close at all”) to 7 (“very close”). For each of the four SCM characters, participants were asked to choose the overlapping circles that best described their relationship with the character presented to the right side of the picture. We included the IOS scale as a manipulation check for whether participants identified with the student character as their implicit in-group more than with the out-group characters.

The second set of the block asked participants to assess the perceived warmth and competence of each of the four SCM characters. This part served as a quick manipulation check of the SCM characters, namely that they fell in their respective quadrants of the SCM two-dimensional space. Warmth and competence ratings occurred on two scales. The first scale assessed warmth and competence (on a Likert scale from 1 to 7; see Measured variables for more details) based on how the respondents believe others perceive the targets (“Please indicate how you believe the Swiss society perceives a typical [student / elder / businessperson / alcoholic]”). The second scale assessed warmth and competence based on how the respondents themselves perceived the SCM targets (“Please indicate how you perceive a typical [student / elder / businessperson / alcoholic]”). These two scales (how the society perceives the characters and how I perceive them) were also presented in random order and had 8 adjectives randomly presented (4 adjectives for warmth and 4 adjectives for competence, in line with ^12^ and ^13^). The final warmth score was the average of ratings for the adjectives “warmhearted” (“warmherzig”), “likeable” (“liebenswert”), “friendly” (“freundlich”), “trustworthy” (“vertrauenswürdig”). The final competence score was the average of ratings for the adjectives “self-confident” (“selbstbewusst”), “intelligent” (“intelligent”), “knowledgeable” (“sachkundig”), “competent” (“kompetent”). For each of the IOS and warmth/competence ratings, respondents saw the instructions (e.g. “Please indicate how close you feel to a typical [student / elder / businessperson / alcoholic]”) in the middle of the screen, a picture of the SCM target character at the top of the screen and the simultaneous 8 adjectives at the bottom of the screen (listed in random order).

The third block of the experiment asked respondents to rate each of the forty-eight scenarios on six attributes (sociality, valence, frequency, controllability, emotional intensity and personal experience), using a VAS. For each situation, a text describing the situation was presented at the top of the page. Sociality was assessed by asking “Please indicate the extent to which the situation only concerns the person depicted or other person(s) as well” (“Geben Sie bitte an, in welchem Ausmass die Situation lediglich die dargestellte Person betrifft oder auch (eine) andere Person(en)”) using a VAS from -50 (“concerns only the individual”/”betrifft nur das Individuum”) to 50 (“concerns other person(s)”/”betrifft auch (eine) andere Person(en)”). Valence was assessed by asking “How NEGATIVE or POSITIVE do you find this situation?” (“Wie NEGATIV bzw. POSITIV empfinden Sie diese Situation?”) using a VAS from -50 (“very negative”/”sehr negativ”) to 50 (“very positive”/”sehr positiv”). Frequency was assessed by asking “How RARE or FREQUENT do you think this situation occurs in everyday life?” (“Wie SELTEN bzw. HÄUFIG tritt diese Situation Ihrer Meinung nach im Alltag auf?”) using a VAS from 0 (“very rare”/”sehr selten”) to 100 (“very often”/”sehr häufig”). Controllability was assessed by asking “How much CONTROL do you think one has over this situation?” (“ Wie viel KONTROLLE hat man Ihrer Meinung nach über diese Situation?”) using a VAS from 0 (“little control”/”wenig Kontrolle”) to 100 (“a lot of control”/”viel Kontrolle”). Emotional intensity was assessed by asking “How EMOTIONALLY INTENSE do you think this situation is?” (“Wie INTENSIV EMOTIONAL ist diese Situation Ihrer Meinung nach?”) using a VAS from 0 (“not at all emotional”/”gar nicht emotional”) to 100 (“very emotional”/”sehr emotional”). Finally, information about the personal experience with the situations was assessed by asking “How much EXPERIENCE have you already had with this situation in the past?” (“Wie viel ERFAHRUNG haben Sie bereits mit dieser Situation in der Vergangenheit gesammelt?”) using a VAS from 0 (“no experience at all”/”gar keine Erfahrung”) to 100 (“a lot of experience”/“sehr viel Erfahrung”).

The fourth and final block of the experiment asked participants to take the following questionnaires: the short version of the Attachment Scale Questionnaire (ASQ; ^14^), Life Orientation Test-Revised (LOT-R; ^15^), Comparative Optimism Scale ^16^, the short Big Five ^17^, the Narcissism Personality Inventory (NPI; ^18^), and the Narcissism Inventory (NI-20; ^19^). In this manuscript, only the data from the ASQ was analyzed.

## S4 Analysis: Design and data cleaning

We manipulated four within-subjects variables: two factors pertaining to the scenarios (valence: desirable and undesirable; sociality: alone and social) and two factors pertaining to the SCM characters (warmth: warm and cold; competence: competent and incompetent). Our dependent variable was the likelihood estimate of a character experiencing a target situation.

We used linear mixed modeling to test our hypotheses, as implemented in the GAMLj module in jamovi (The jamovi project (2020). jamovi. (Version 1.2.27) [Computer Software]. Retrieved from https://www.jamovi.org.). The design was completely crossed, with Subjects (Level 2 data) crossed with four SCM characters presented in identical 48 situations (both SCM characters and the situations are Level 1 data because all participants will see all of the 4 x 48 combinations). The Level 1 outcome was the likelihood estimate and the Level 1 predictors were the SCM warmth (warm vs. cold) and SCM competence (competent vs. incompetent) of the character, the sociality of the situation (alone vs. social) and the valence of the situation (desirable vs. undesirable). The Level 2 predictors were the participants’ scores of attachment anxiety and attachment avoidance. The software Jamovi centers by default the continuous predictors around the mean.

*Data cleaning.* In line with our previous study ^9^, outlier participants were identified and excluded from the analysis if their answers of 0%, 50% or 100% represented more than three standard deviations above the same answers at the sample level. Specifically, we first calculated the percentages that the likelihood estimates of 0%, 50% and 100% received out of the total estimates at the level of each participant and then at the sample level (i.e. averaged across participants). At sample level, we then additionally calculated the standard deviations of the percentages that the likelihood estimates of 0%, 50% and 100% received. Next, we flagged those participants whose percentages of either 0%, 50% or 100% likelihood estimates were more than three standard deviations above the percentages of estimates at the sample level.

Following this procedure, we identified eight outliers: **three** participants repeatedly chose the default scale value of 50% (16%, 22% and 17% of their total answers, respectively), **two** participants frequently chose the 0% likelihood estimates (14% and 19% of the trials, respectively), **one** participant frequently chose the 100% likelihood estimate (34% of the total answers) and **two** participants repeatedly chose both likelihood estimates of 0% (22% and 19% of their answers, respectively) and 100% (42% and 33% of all their answers, respectively). All analyses reported below were conducted with the remaining sample of 194 participants. We note that running the analyses with and without the outlier participants led to similar findings.

# S5: Model selection.

Both participants and scenarios had random intercepts and we selected the model that best fit the data after a comparison of models with and without random slopes ^20,21^.

**Final linear mixed model for likelihood estimates as Level 1 outcome**

| Model Info | | | |
| --- | --- | --- | --- |
|  |  |  |  |
| Estimate |  | Linear mixed model fit by REML |  |
| Call |  | Likelihood estimate ~ 1 + Warmth + Competence + Sociality + Valence + Attachment anxiety + Attachment avoidance + Warmth:Competence + Warmth:Sociality + Competence:Sociality + Warmth:Valence + Competence:Valence + Sociality:Valence + Warmth:Attachment anxiety + Sociality:Attachment anxiety + Valence:Attachment anxiety + Warmth:Attachment avoidance + Sociality:Attachment avoidance + Valence:Attachment avoidance + Attachment avoidance:Competence + Warmth:Competence:Sociality + Warmth:Competence:Valence + Warmth:Sociality:Valence + Competence:Sociality:Valence + Warmth:Sociality:Attachment anxiety + Warmth:Valence:Attachment anxiety + Sociality:Valence:Attachment anxiety + Warmth:Sociality:Attachment avoidance + Warmth:Valence:Attachment avoidance + Sociality:Valence:Attachment avoidance + Warmth:Attachment avoidance:Competence + Sociality:Attachment avoidance:Competence + Valence:Attachment avoidance:Competence + Warmth:Competence:Sociality:Valence + Warmth:Sociality:Valence:Attachment anxiety + Warmth:Sociality:Valence:Attachment avoidance + Warmth:Sociality:Attachment avoidance:Competence + Warmth:Valence:Attachment avoidance:Competence + Sociality:Valence:Attachment avoidance:Competence + Warmth:Sociality:Valence:Attachment avoidance:Competence+( 1 \| Scenario_ID )+( 1 + Valence + Warmth + Competence \| Subject_ID ) |  |
| AIC |  | 339863.518 |  |
| BIC |  | 340242.630 |  |
| LogLikel. |  | -169847.656 |  |
| R-squared Marginal |  | 0.114 |  |
| R-squared Conditional |  | 0.325 |  |
| Converged |  | Yes |  |

| Fixed Effect Omnibus tests | | | | | | | | | |
| --- | --- | --- | --- | --- | --- | --- | --- | --- | --- |
|  |  |  |  |  |  |  |  |  |  |
|  | | **F** | | **Num df** | | **Den df** | | **p** | |
| Warmth |  | 217.7975 |  | 1 |  | 191.8 |  | < .001 |  |
| Competence |  | 546.6081 |  | 1 |  | 192.1 |  | < .001 |  |
| Sociality |  | 0.4312 |  | 1 |  | 44.0 |  | 0.515 |  |
| Valence |  | 0.4879 |  | 1 |  | 45.9 |  | 0.488 |  |
| Attachment anxiety |  | 4.0965 |  | 1 |  | 191.0 |  | 0.044 |  |
| Attachment avoidance |  | 0.2585 |  | 1 |  | 193.1 |  | 0.612 |  |
| Warmth ✻ Competence |  | 0.4254 |  | 1 |  | 36406.0 |  | 0.514 |  |
| Warmth ✻ Sociality |  | 5.2452 |  | 1 |  | 36406.0 |  | 0.022 |  |
| Competence ✻ Sociality |  | 34.6622 |  | 1 |  | 36406.0 |  | < .001 |  |
| Warmth ✻ Valence |  | 1778.6130 |  | 1 |  | 36406.0 |  | < .001 |  |
| Competence ✻ Valence |  | 656.7584 |  | 1 |  | 36406.0 |  | < .001 |  |
| Sociality ✻ Valence |  | 0.0272 |  | 1 |  | 44.0 |  | 0.870 |  |
| Warmth ✻ Attachment anxiety |  | 1.6503 |  | 1 |  | 194.8 |  | 0.200 |  |
| Sociality ✻ Attachment anxiety |  | 0.2957 |  | 1 |  | 36406.0 |  | 0.587 |  |
| Valence ✻ Attachment anxiety |  | 0.5187 |  | 1 |  | 194.3 |  | 0.472 |  |
| Sociality ✻ Attachment avoidance |  | 0.2131 |  | 1 |  | 36406.0 |  | 0.644 |  |
| Valence ✻ Attachment avoidance |  | 1.9534 |  | 1 |  | 194.6 |  | 0.164 |  |
| Competence ✻ Attachment avoidance |  | 0.1929 |  | 1 |  | 194.6 |  | 0.661 |  |
| Warmth ✻ Competence ✻ Sociality |  | 105.6641 |  | 1 |  | 36406.0 |  | < .001 |  |
| Warmth ✻ Competence ✻ Valence |  | 957.1694 |  | 1 |  | 36406.0 |  | < .001 |  |
| Warmth ✻ Sociality ✻ Valence |  | 187.2213 |  | 1 |  | 36406.0 |  | < .001 |  |
| Competence ✻ Sociality ✻ Valence |  | 88.6159 |  | 1 |  | 36406.0 |  | < .001 |  |
| Warmth ✻ Sociality ✻ Attachment anxiety |  | 0.3522 |  | 1 |  | 36406.0 |  | 0.553 |  |
| Warmth ✻ Valence ✻ Attachment anxiety |  | 0.3753 |  | 1 |  | 36406.0 |  | 0.540 |  |
| Sociality ✻ Valence ✻ Attachment anxiety |  | 1.7501 |  | 1 |  | 36406.0 |  | 0.186 |  |
| Sociality ✻ Valence ✻ Attachment avoidance |  | 0.2316 |  | 1 |  | 36406.0 |  | 0.630 |  |
| Competence ✻ Sociality ✻ Attachment avoidance |  | 0.4061 |  | 1 |  | 36406.0 |  | 0.524 |  |
| Competence ✻ Valence ✻ Attachment avoidance |  | 6.0355 |  | 1 |  | 36406.0 |  | 0.014 |  |
| Warmth ✻ Competence ✻ Sociality ✻ Valence |  | 55.8399 |  | 1 |  | 36406.0 |  | < .001 |  |
| Warmth ✻ Sociality ✻ Valence ✻ Attachment anxiety |  | 0.3257 |  | 1 |  | 36406.0 |  | 0.568 |  |
| Competence ✻ Sociality ✻ Valence ✻ Attachment avoidance |  | 0.1456 |  | 1 |  | 36406.0 |  | 0.703 |  |
| Note. Satterthwaite method for degrees of freedom | | | | | | | | | |
|  | | | | | | | | | |

**Comparison of linear mixed models with likelihood estimate as Level 1 predictor**

Model 1: random intercepts only for participants and scenarios

Model 2: random intercepts for participant and scenario + fixed effects for warmth, competence, valence, sociality, attachment anxiety, attachment avoidance

Model 3: random intercepts for participant and scenario + fixed effects for warmth, competence, valence, sociality, attachment anxiety, attachment avoidance as well as their 2-way, 3-way, 4-way and 5-way interactions

Model 4: random intercepts for participant and scenario + fixed effects for warmth, competence, valence, sociality, attachment anxiety, attachment avoidance as well as their 2-way, 3-way, 4-way and 5-way interactions + random slope for valence

Model 5: random intercepts for participant and scenario + fixed effects for warmth, competence, valence, sociality, attachment anxiety, attachment avoidance as well as their 2-way, 3-way, 4-way and 5-way interactions + random slope for warmth

Model 6: random intercepts for participant and scenario + fixed effects for warmth, competence, valence, sociality, attachment anxiety, attachment avoidance as well as their 2-way, 3-way, 4-way and 5-way interactions + random slope for competence

Model 7: random intercepts for participant and scenario + fixed effects for warmth, competence, valence, sociality, attachment anxiety, attachment avoidance as well as their 2-way, 3-way, 4-way and 5-way interactions + random slope for valence, warmth

Model 8: random intercepts for participant and scenario + fixed effects for warmth, competence, valence, sociality, attachment anxiety, attachment avoidance as well as their 2-way, 3-way, 4-way and 5-way interactions + random slope for valence, competence

Model 9: random intercepts for participant and scenario + fixed effects for warmth, competence, valence, sociality, attachment anxiety, attachment avoidance as well as their 2-way, 3-way, 4-way and 5-way interactions + random slope for warmth, competence

Model 10: random intercepts for participant and scenario + fixed effects for warmth, competence, valence, sociality, attachment anxiety, attachment avoidance as well as their 2-way, 3-way, 4-way and 5-way interactions + random slope for valence, warmth, competence

| Likelihood estimates | AIC | BIC | LogLikelihood | Parameters |
| --- | --- | --- | --- | --- |
| Model 1 | 433926.930 | 433959.072 | -216958.039 | 4 = 1 + 3 |
| Model 2 attachment | 344086.7518 | 344161.1786 | --172027.9625 | 10 = 7 + 3 |
| Model 3 interactions | 340540.337 | 340798.930 | -170215.271 | 35 = 32 + 3 |
| Model 4 slope valence | 340152.913 | 340425.968 | -170018.265 | 36 = 32 + 4 |
| Model 5 slope warmth | 340452.036 | 340726.175 | -170168.369 | 36 = 32 + 4 |
| Model 6 slope competence | 340383.291 | 340657.191 | -170133.876 | 36 = 32 + 4 |
| Model 7 slope warmth, competence | 340284.469 | 340582.561 | -170080.774 | 36 = 32 + 5 |
| Model 8 slope valence, warmth | 340042.504 | 340339.788 | -169959.387 | 37 = 32 + 5 |
| Model 9 slope valence, competence | 339969.679 | 340266.744 | -169922.865 | 37 = 32 + 5 |
| Model 10 slope valence, warmth, competence | 339854.901 | 340184.773 | -169860.829 | 38 = 32 + 6 |

**Supplementary Table S5**. Comparison of eight models using restricted maximum likelihood (REML) and assuming correlation between variances of random effects.

**S6: Exploratory analyses**

| Model Info | | | |
| --- | --- | --- | --- |
|  |  |  |  |
| **Info** | |  | |
| Estimate |  | Linear mixed model fit by REML |  |
| Call |  | Likelihood estimate ~ 1 + Valence + Sociality + Attachment anxiety + Attachment avoidance + IOS + Valence:Sociality + Valence:IOS + Sociality:IOS + Valence:Attachment anxiety + Sociality:Attachment anxiety + IOS:Attachment anxiety + Valence:Attachment avoidance + Sociality:Attachment avoidance + IOS:Attachment avoidance + Valence:Sociality:IOS + Valence:Sociality:Attachment anxiety + Valence:IOS:Attachment anxiety + Sociality:IOS:Attachment anxiety + Valence:Sociality:Attachment avoidance + Valence:IOS:Attachment avoidance + Sociality:IOS:Attachment avoidance + Valence:Sociality:IOS:Attachment anxiety + Valence:Sociality:IOS:Attachment avoidance+( 1 \| Scenario_ID )+( 1 + Valence \| Subject_ID ) |  |
| AIC |  | 343482.4402 |  |
| BIC |  | 343721.8409 |  |
| LogLikel. |  | -171708.3028 |  |
| R-squared Marginal |  | 0.0547 |  |
| R-squared Conditional |  | 0.2541 |  |
| Converged |  | Yes |  |
|  | | | |

| Fixed Effect Omnibus tests | | | | | | | | | |
| --- | --- | --- | --- | --- | --- | --- | --- | --- | --- |
|  |  |  |  |  |  |  |  |  |  |
|  | | **F** | | **Num df** | | **Den df** | | **p** | |
| Valence |  | 0.5055 |  | 1 |  | 46.0 |  | 0.481 |  |
| Sociality |  | 0.4234 |  | 1 |  | 44.0 |  | 0.519 |  |
| Attachment anxiety |  | 1.1283 |  | 1 |  | 190.8 |  | 0.289 |  |
| Attachment avoidance |  | 0.5505 |  | 1 |  | 190.8 |  | 0.459 |  |
| IOS |  | 1057.5176 |  | 1 |  | 36157.0 |  | < .001 |  |
| Valence ✻ Sociality |  | 0.0274 |  | 1 |  | 44.0 |  | 0.869 |  |
| Valence ✻ IOS |  | 1062.0311 |  | 1 |  | 30199.1 |  | < .001 |  |
| Sociality ✻ IOS |  | 29.4415 |  | 1 |  | 36796.7 |  | < .001 |  |
| Valence ✻ Attachment anxiety |  | 3.9312 |  | 1 |  | 189.7 |  | 0.049 |  |
| Sociality ✻ Attachment anxiety |  | 0.5564 |  | 1 |  | 36796.7 |  | 0.456 |  |
| Attachment anxiety ✻ IOS |  | 4.8065 |  | 1 |  | 35904.1 |  | 0.028 |  |
| Valence ✻ Attachment avoidance |  | 1.1251 |  | 1 |  | 189.7 |  | 0.290 |  |
| Sociality ✻ Attachment avoidance |  | 0.1676 |  | 1 |  | 36796.7 |  | 0.682 |  |
| Attachment avoidance ✻ IOS |  | 9.4528 |  | 1 |  | 36414.5 |  | 0.002 |  |
| Valence ✻ Sociality ✻ IOS |  | 28.0545 |  | 1 |  | 36796.7 |  | < .001 |  |
| Valence ✻ Sociality ✻ Attachment anxiety |  | 1.0343 |  | 1 |  | 36796.7 |  | 0.309 |  |
| Valence ✻ Attachment anxiety ✻ IOS |  | 7.8578 |  | 1 |  | 28958.5 |  | 0.005 |  |
| Sociality ✻ Attachment anxiety ✻ IOS |  | 3.0043 |  | 1 |  | 36796.7 |  | 0.083 |  |
| Valence ✻ Sociality ✻ Attachment avoidance |  | 0.1624 |  | 1 |  | 36796.7 |  | 0.687 |  |
| Valence ✻ Attachment avoidance ✻ IOS |  | 4.0721 |  | 1 |  | 31615.8 |  | 0.044 |  |
| Sociality ✻ Attachment avoidance ✻ IOS |  | 2.5906 |  | 1 |  | 36796.7 |  | 0.108 |  |
| Valence ✻ Sociality ✻ Attachment anxiety ✻ IOS |  | 0.0481 |  | 1 |  | 36796.7 |  | 0.826 |  |
| Valence ✻ Sociality ✻ Attachment avoidance ✻ IOS |  | 0.1922 |  | 1 |  | 36796.7 |  | 0.661 |  |
| Note. Satterthwaite method for degrees of freedom | | | | | | | | | |
|  | | | | | | | | | |

**References**

1 Cuddy, A. J., Fiske, S. T. & Glick, P. The BIAS map: behaviors from intergroup affect and stereotypes. *Journal of personality and social psychology* **92**, 631 (2007).

2 Castano, E., Yzerbyt, V., Bourguignon, D. & Seron, E. Who may enter? The impact of in-group identification on in-group/out-group categorization. *Journal of Experimental Social Psychology* **38**, 315-322 (2002).

3 Harris, P., Middleton, W. & Joiner, R. The typical student as an in‐group member: eliminating optimistic bias by reducing social distance. *Eur. J. Soc. Psychol.* **30**, 235-253 (2000).

4 Hogg, M. A. & Reid, S. A. Social identity, self‐categorization, and the communication of group norms. *Communication Theory* **16**, 7-30 (2006).

5 Reynolds, K. J., Turner, J. C. & Haslam, S. A. When are we better than them and they worse than us? A closer look at social discrimination in positive and negative domains. *Journal of Personality and Social Psychology* **78**, 64 (2000).

6 Cuddy, A. J., Norton, M. I. & Fiske, S. T. This old stereotype: The pervasiveness and persistence of the elderly stereotype. *J. Soc. Issues* **61**, 267-285 (2005).

7 Fiske, S. T. Envy up, scorn down: How comparison divides us. *American Psychologist* **65**, 698 (2010).

8 Fiske, S. T. Divided by status: Upward envy and downward scorn. *Proceedings of the American Philosophical Society* **157**, 261 (2013).

9 Dricu, M. *et al.* Warmth and competence predict overoptimistic beliefs for out-group but not in-group members. *PloS one* **13**, e0207670 (2018).

10 Aron, A., Aron, E. N. & Smollan, D. Inclusion of other in the self scale and the structure of interpersonal closeness. *Journal of personality and social psychology* **63**, 596 (1992).

11 Gächter, S., Starmer, C. & Tufano, F. Measuring the closeness of relationships: a comprehensive evaluation of the'inclusion of the other in the self'scale. *PloS one* **10**, e0129478 (2015).

12 Kervyn, N., Fiske, S. & Yzerbyt, V. Forecasting the primary dimension of social perception. *Social Psychology* (2015).

13 Leach, C. W., Ellemers, N. & Barreto, M. Group virtue: the importance of morality (vs. competence and sociability) in the positive evaluation of in-groups. *Journal of Personality and Social Psychology* **93**, 234 (2007).

14 Karantzas, G. C., Feeney, J. A. & Wilkinson, R. Is less more? Confirmatory factor analysis of the Attachment Style Questionnaires. *Journal of Social and Personal Relationships* **27**, 749-780 (2010).

15 Scheier, M. F., Carver, C. S. & Bridges, M. W. Distinguishing optimism from neuroticism (and trait anxiety, self-mastery, and self-esteem): a reevaluation of the Life Orientation Test. *Journal of personality and social psychology* **67**, 1063 (1994).

16 Weinstein, N. D. Unrealistic optimism about future life events. *Journal of personality and social psychology* **39**, 806 (1980).

17 Rammstedt, B., Danner, D., Soto, C. J. & John, O. P. Validation of the short and extra-short forms of the Big Five Inventory-2 (BFI-2) and their German adaptations. *European Journal of Psychological Assessment* (2018).

18 Schütz, A., Marcus, B. & Sellin, I. Die Messung von Narzissmus als Persönlichkeitskonstrukt. *Diagnostica* **50**, 202-218 (2004).

19 Daig, I. *et al.* Development and factorial validation of a short version of the narcissism inventory (NI-20). *Psychopathology* **43**, 150-158 (2010).

20 Gurka, M. J. Selecting the best linear mixed model under REML. *The American Statistician* **60**, 19-26 (2006).

21 Aguinis, H., Gottfredson, R. K. & Culpepper, S. A. Best-practice recommendations for estimating cross-level interaction effects using multilevel modeling. *Journal of Management* **39**, 1490-1528 (2013).
